# Urinary metabolomics identifies isoleucine as a prognostic biomarker for progression of diabetic kidney disease

**DOI:** 10.3389/fendo.2026.1787886

**Published:** 2026-03-23

**Authors:** Yufei Li, Donghwan Yun, Soo Heon Kwak, Seung Seok Han, Joo-Youn Cho

**Affiliations:** 1 Department of Clinical Pharmacology and Therapeutics, Seoul National University College of Medicine and Hospital, Seoul, Republic of Korea; 2 Division of Nephrology, Seoul National University College of Medicine, Seoul, Republic of Korea; 3 Division of Endocrinology and Metabolism, Seoul National University College of Medicine and Seoul National University Hospital, Seoul, Republic of Korea; 4 Department of Biomedical Sciences, Seoul National University College of Medicine, Seoul, Republic of Korea; 5 Kidney Research Institute, Seoul National University Medical Research Center, Seoul, Republic of Korea

**Keywords:** chronic kidney disease, diabetic kidney disease, disease progression, prognostic biomarker, urinary metabolomics

## Abstract

**Background:**

Diabetic kidney disease (DKD), a major microvascular complication of diabetes mellitus, is the leading cause of chronic kidney disease and end-stage kidney disease worldwide. However, current clinical markers are limited in sensitivity for early detection and prognosis.

**Methods:**

We recruited 96 patients with biopsy-confirmed diabetic nephropathy based on type 2 diabetes (T2DN), 76 patients with type 2 diabetes (T2D), and 79 healthy controls (HC). Untargeted urinary metabolomics was performed, followed by pathway and network analyses. Prognostic metabolites were identified using Cox regression and Kaplan–Meier survival analyses adjusted for confounders. Associations with renal histopathology were evaluated using partial Spearman correlation. For key prognostic metabolites, targeted quantification was performed in urine and plasma for validation.

**Results:**

Among 148 detected metabolites, 101 significantly differed across groups, spanning multiple metabolic pathways. Amino acid metabolism, particularly branched-chain amino acid pathways, showed progressive changes along the HC–T2D–T2DN continuum. Urinary isoleucine emerged as the top prognostic metabolite (2.18 [1.18, 4.03], *P* = 0.013), stratifying renal survival in T2DN (log-rank *P* < 0.001) and correlating with four renal histological scores, which together capture the extent of renal structural damage and disease progression. Targeted quantification confirmed these findings and demonstrated that urinary, but not plasma, isoleucine was prognostically relevant.

**Conclusions:**

Urinary isoleucine is a potential non-invasive biomarker of DKD progression, reflecting tubular injury and underlying metabolic dysregulation. These findings support urinary metabolomics as a tool for prognostic risk stratification, with the potential to enable future precision clinical management in DKD.

## 1 Introduction

Diabetes mellitus is a major and growing global health concern, with prevalence continuing to rise and substantially contributing to morbidity and mortality worldwide (1). Among its complications, diabetic kidney disease (DKD) has become the leading cause of chronic kidney disease (CKD) and a major driver of end-stage kidney disease (ESKD), imposing significant healthcare and socioeconomic burdens (2, 3). Although DKD is strongly associated with chronic hyperglycemia and insulin resistance, its pathogenesis remains incompletely understood. Notably, a substantial proportion of individuals with diabetes develop nephropathy despite optimal glycemic and blood pressure control, reflecting marked clinical heterogeneity (4–7), which highlights the urgent need for more sensitive, mechanism-informed tools to identify high-risk individuals and more effectively monitor disease progression, particularly in the early, subclinical stages when intervention is still feasible (8, 9).

To address this need, recognizing the limitations of current diagnostic and monitoring strategies for DKD is important. Currently, clinical evaluation primarily relies on estimated Glomerular filtration rate (eGFR) and urine albumin-to-creatinine ratio (UACR), which reflect glomerular filtration and barrier integrity (10). However, these markers are often insensitive to early or subclinical tubular injury, which is increasingly recognized as a major contributor to DKD progression. Although renal biopsy remains the gold standard for definitive diagnosis and provides valuable histopathological insights, it is invasive and impractical for routine use (11). Several tubular injury markers, including neutrophil gelatinase-associated lipocalin (NGAL) and kidney injury molecule-1 (KIM-1), have been proposed; however, their clinical utility remains limited owing to inconsistent performance, lack of standardized assays, and insufficient validation in large, diverse cohorts (12).

Metabolomics offers a powerful approach for characterizing the metabolic dysregulation underlying kidney diseases, enabling comprehensive profiling of small molecular metabolites across both systemic and organ-specific contexts (13). Although metabolomics can be applied to various biological specimens, urine is particularly valuable for kidney disease research, as it reflects renal tubular processing and may more accurately capture localized metabolic changes (13). Further, compared to blood-based metabolomics, urinary profiling offers a non-invasive, easily accessible, and repeatable approach for longitudinal monitoring. However, most existing metabolomics studies have focused on plasma or serum and predominantly relied on correlation-based analyses, limiting both mechanistic interpretability and tissue specificity (14–16).

Based on these considerations, we hypothesized that urinary metabolomic profiles considerably differ among healthy controls (HC), patients with type 2 diabetes (T2D), and patients with type 2 diabetic nephropathy (T2DN). We further posited that certain urinary metabolites may serve as non-invasive prognostic biomarkers of DKD progression. To test these hypotheses, we conducted comprehensive urinary metabolomic profiling, coupled with pathway analysis, survival modeling, and histopathological correlation, to identify metabolites with both prognostic utility and mechanistic relevance in DKD.

## 2 Materials and methods

### 2.1 Participants and sample collection

Between December 2011 and November 2022, 96 patients with biopsy-proven T2DN, 76 patients with T2D, and 79 healthy adults were enrolled at Seoul National University Hospital. All human biospecimens were collected under written informed consent approved by the Institutional Review Board of Seoul National University Hospital (IRB No. H-2104-120-1214). For patients with biopsy-proven T2DN, informed consent for biospecimen collection and future secondary research use was obtained on the day of kidney biopsy. For participants in the T2D control group, informed consent was obtained during outpatient clinic visits at the time of biospecimen collection.

T2DN was defined as histopathological confirmation of diabetic nephropathy as the primary diagnosis on kidney biopsy, with no evidence of concomitant primary or secondary glomerulonephritis. The T2D group comprised endocrinology outpatients with preserved kidney function (eGFR > 60 mL/min/1.73 m²) and without evidence of proteinuria, defined based on repeated random urine protein-to-creatinine ratio (PCR) measurements obtained during outpatient visits to confirm the absence of persistent proteinuria prior to enrollment. Healthy adults, defined as having no diabetes or other known chronic diseases, served as the HC group.

Clinical and laboratory data, along with urine samples, were collected immediately before biopsy or at the time of biospecimen donation. Urine was obtained via midstream clean-catch, processed immediately to minimize metabolite degradation, and centrifuged at 3,000 rpm for 10 min to remove cellular debris. The supernatant was aliquoted into cryovials, promptly frozen, and stored at -80 °C before use. Among the patients with T2DN, baseline plasma samples and genome-wide association study (GWAS) data were available for 64 individuals, as previously reported (17).

### 2.2 Chemicals and reagents

High performance liquid chromatography (HPLC) grade acetonitrile, methanol, ethanol, and water were purchased from J.T. Baker (Phillipsburg, NJ, USA). High-purity L-leucine (≥98%), L-isoleucine (≥98%), formic acid (≥95%), and N-trimethylsilyl-N-methyl trifluoroacetamide (MSTFA, ≥98.5%) were purchased from Sigma-Aldrich (St. Louis, MO, USA).

### 2.3 Untargeted metabolomics analysis

Frozen urine samples were thawed at 4 °C before analysis. Quality control (QC) samples were prepared by pooling small aliquots from all individual samples. For metabolite extraction, 50 μL of each sample or QC was mixed with 200 μL of cold (-20 °C) methanol:ethanol (1:1, v/v) in fresh tubes. The mixtures were vortexed for 5 min and subsequently centrifuged at 14,000 rpm for 10 min at 4 °C to precipitate proteins. The resulting supernatants were transferred into autosampler vials for subsequent analysis.

Global metabolite profiling was performed using a Vanquish Flex ultra-HPLC system coupled with an Orbitrap Exploris 120 mass spectrometer (Thermo Fisher Scientific). Chromatographic separation was achieved using an ACQUITY UPLC HSS T3 column (2.1 × 100 mm, 1.8 μm; Waters). The mobile phases comprised solvents A (0.1% formic acid in water) and B (0.1% formic acid in acetonitrile:methanol, 7:3). The gradient started at 5% B for 1 min, linearly increased to 80% B over 12 min, was maintained at 80% B for 0.5 min, and subsequently returned to 5% B at 13.5 min with a total runtime of 15 min. The flow rate was set to 0.4 mL/min. The column oven temperature was maintained at 40 °C, and the autosampler was kept at 4 °C, with an injection volume of 4 μL. Sample injections were randomized, and QC samples were analyzed after every 10 runs.

Mass spectrometric data were acquired in both positive and negative ionization modes and processed separately. Full MS scans were performed for all samples, whereas data-dependent MS/MS (ddMS2) fragmentation was carried out only for QC samples using stepped collision energies ranging from 10 to 50 eV, with four dependent scans collected per ddMS2 cycle. Data processing was performed using the TidyMass R package (version 1.0.8) (18). Features with >20% missing values in QC samples or >50% missing values across all three groups were filtered to ensure data quality. The remaining missing values were imputed using the k-nearest neighbors algorithm. Metabolite annotation was performed by matching MS2 spectra against mass spectral databases (Human Metabolome Database, MassBank, MassBank of North America) and two in-house libraries provided by TidyMass. Finally, data from the positive and negative ionization modes were merged, and endogenous metabolite features were retained for downstream analyses.

### 2.4 Targeted leucine and isoleucine quantitation

For sample preparation, 50 μL of each calibrator or biological sample was mixed with 200 μL of pre-chilled methanol:ethanol (1:1, v/v) in a new tube. The mixture was vortexed for 5 min, followed by centrifugation at 14,000 rpm for 10 min at 4 °C to remove protein precipitates and other debris. A total of 50 μL of the supernatant was transferred to a new tube and evaporated to dryness using a nitrogen evaporator. Each dried sample was derivatized with 100 μL of MSTFA at 30 °C for 30 min, and subsequently transferred to an autosampler vial containing a glass insert for analysis.

Quantitative determination of isoleucine and leucine was performed via an Agilent 7890B gas chromatograph coupled to a 7000B triple quadrupole mass spectrometer (Agilent Technologies Inc., Santa Clara, CA, USA) with an HP-5MS capillary column (30 m × 0.25 mm, 0.25 μm film thickness; Supelco). Calibration standards and QC samples were prepared by serial dilution of stock solutions in methanol. Eight-point calibration curves were generated for each analyte, with final concentration ranges of 0.2–256 µg/mL for leucine and 0.1–128 µg/mL for isoleucine.

A 1-μL aliquot of each sample was injected in splitless mode. The oven temperature program was as follows: initially held at 80 °C for 1 min, ramped to 280 °C at 20 °C/min, and held at 280 °C for 5 min. High-purity helium (99.99%) was used as the carrier gas at a constant flow rate of 1.5 mL/min. Multiple reaction monitoring was performed using a transition from m/z 158 to 73 with a collision energy of 10 eV for both isoleucine and leucine. The two analytes were separated based on their retention times (leucine: 5.105 min; isoleucine: 5.227 min). Calibration curves showed excellent linearity (R² > 0.99), and QC samples demonstrated good analytical reproducibility (CV < 15%).

### 2.5 Statistics and visualization

Urinary dilution is commonly corrected using creatinine; however, this approach is inappropriate in CKD due to impaired and variable creatinine excretion. For untargeted metabolomics, probabilistic quotient normalization was applied using a reference spectrum defined as the median of all samples. The median quotient of each sample relative to the reference spectrum across all detected features was used as the sample-specific normalization factor. For targeted quantitative analyses, urine specific gravity (SG) was measured using a refractometer, and analyte concentrations were adjusted according to the following formula: corrected concentration = raw concentration × (median SG − 1)/(sample SG − 1), where the cohort median SG (1.0165) served as the reference value.

All statistical analyses and data visualizations described below, unless otherwise specified, were performed using R (version 4.3.3). Inter-sample variability and clustering patterns were assessed using principal component analysis (PCA), with the first two principal components (PC1 and PC2) used for visualization. Unsupervised hierarchical clustering heatmaps were generated using Euclidean distance and Ward’s method to visualize metabolite expression patterns. The Kruskal–Wallis test was used for multi-group analyses, followed by Dunn’s *post hoc* test for pairwise comparisons. Because sex, age, and body mass index (BMI) showed significant differences across groups, they were included as covariates to control for potential confounding effects in subsequent analyses. Benjamini–Hochberg false discovery rate (FDR) correction was applied, with an FDR < 0.05 considered statistically significant. A volcano plot was generated using FDR values and fold changes to visualize differentially expressed metabolites across comparisons. To illustrate biochemical relationships among significant metabolites, network analysis was performed using MetaMapp to cluster metabolites based on structural similarity, and the resulting metabolic network was visualized in Cytoscape (19). Pathway enrichment analysis was conducted via MetaboAnalyst with *P* < 0.05 as the significance threshold (20).

To evaluate potential storage-related variability due to the extended inclusion period, storage duration was calculated for each sample as the time between collection and metabolomic analysis. Group differences were assessed using the Kruskal–Wallis test. Associations between storage duration and key metabolites were examined using Spearman correlation. In addition, storage duration was included as an additional covariate in sensitivity Cox regression analyses.

### 2.6 Survival analysis

For the T2DN subgroup, primary renal outcomes were longitudinally collected to reflect DKD progression and were defined as the occurrence and timing of any of the following three events (1): doubling of serum creatinine, (2) ≥50% decline in eGFR, or (3) progression to ESKD. To assess the prognostic significance of metabolites associated with DKD progression, Cox proportional hazards regression analyses were first performed on the metabolites that showed significant differences across groups. Covariate-adjusted Cox models (age, sex, BMI, and eGFR) were fitted for each of the 101 metabolites, and FDR correction was applied across these analyses. Both univariate and multivariate Cox models were tested to evaluate the prognostic power of each metabolite individually and in combination. Further, a composite risk score was calculated using the β coefficients derived from the multivariate Cox model, and its predictive ability was assessed through adjusted survival analysis. Kaplan–Meier survival analyses were performed to evaluate the prognostic value of each metabolite, stratifying patients into high- and low-expression groups based on the median value. Survival curves were adjusted for age, sex, BMI, and eGFR, with significance assessed using the log-rank test. For quantitatively analyzed metabolites, stratification was based on the optimal cutoff derived from ROC analysis for renal progression using the Youden index. Analyses were performed on log2-transformed concentrations, and if ROC discrimination was not statistically significant, the median value was used for stratification. The proportional hazards assumption was evaluated using Schoenfeld residual tests for each metabolite and for the composite risk score. In sensitivity analyses, specific gravity was additionally included as a covariate to evaluate potential residual dilution effects.

### 2.7 Correlation analysis

Partial Spearman correlation analysis was performed to assess the associations between urinary metabolites and renal pathological scores, while adjusting for potential confounding variables, including age, sex, and BMI. Correlations were calculated between selected metabolites and four pathological scores: interstitial fibrosis and tubular atrophy (IFTA), glomerular classification, interstitial inflammation, and arteriolar hyalinosis. Partial correlation coefficients (r) and their corresponding *P* values were used to evaluate both the strength and statistical significance of these relationships, with *P* < 0.05 considered statistically significant. To facilitate interpretation, boxplots or violin plots combined with locally estimated scatterplot smoothing (LOESS) curves were generated to illustrate metabolite distributions across different histological grades.

### 2.8 GWAS of urinary isoleucine and Mendelian randomization analysis

To investigate the causal effect of urinary isoleucine levels on DKD progression, a one-sample Mendelian randomization (MR) analysis was conducted using individual-level genotype and phenotype data from the T2DN group. Autosomal SNPs (chromosomes 1–22) were extracted and underwent quality control filtering using PLINK (version 1.9), including genotype missingness rate (<5%) and minor allele frequency (5%). Population structure was accounted for by calculating the top five PCs after linkage disequilibrium (LD) pruning. A linear regression-based GWAS adjusted for age, sex, BMI, and PC1–PC5 was conducted to identify SNPs associated with urinary isoleucine levels. SNPs with *P* < 0.0001 and F-statistics > 20 were retained as candidate instrumental variables (IVs) and subsequently pruned for independence using LD-based clumping (R^2^ < 0.1, window size 500 kb). Causal effects were estimated using two methods implemented in the OneSampleMR R package: two-stage predictor substitution (TSPS) with a logistic link to estimate causal odds ratios (CORs), and the multiplicative structural mean model (MSMM) using generalized method of moments to estimate causal risk ratios (CRRs). Instrument validity was assessed using Hansen’s J-test. Genotypes were coded as allele dosages assuming an additive genetic model.

## 3 Results

### 3.1 Clinical characteristics

Participant (*n* = 251) clinical characteristics are summarized in **Table 1**. Median age and sex distribution significantly differed across groups, with the diabetes-related groups being older and having a higher proportion of males. Although BMI varied, the differences were not significant (*P* = 0.095). Kidney function parameters markedly differed between groups. The T2DN group had significantly higher serum creatinine levels and lower eGFR than both HCs and patients with T2D (*P* < 0.001). These findings confirmed the presence of overt renal impairment in the T2DN group, providing a clinical context for the subsequent metabolomic analyses. Among 96 patients with T2DN, the median follow-up duration was 25.1 months (IQR 11.6–47.0), during which 50 patients (52%) experienced DKD progression.

**Table 1:** Demographic and clinical characteristics of the study population at baseline.

Characteristic		HC	T2D	T2DN	*P*
*N*		79	76	96	
Age		53.0 [45.0, 59.0]	56.5 [50.8, 65.0]	55.0 [44.0, 64.0]	0.015
Sex (%)	Male	38 (48.1)	53 (69.7)	65 (67.7)	0.008
	Female	41 (51.9)	23 (30.3)	31 (32.3)	
BMI		24.0 [21.7, 26.2]	25.2 [23.1, 27.1]	25.2 [22.1, 27.1]	0.095
BUN		14.0 [12.0, 17.0]	13.0 [11.0, 16.0]	26.0 [20.8, 34.2]	<0.001
Creatinine		0.8 [0.7, 0.9]	0.8 [0.6, 0.8]	1.6 [1.2, 2.2]	<0.001
eGFR		97.0 [90.8, 103.8]	99.8 [93.5, 106.0]	43.6 [28.4, 64.6]	<0.001
uPCR		0.0 [0.0, 0.0]	0.0 [0.0, 0.0]	4.5 [2.4, 8.1]	<0.001

HC, healthy controls; T2D, type 2 diabetes without nephropathy; T2DN, type 2 diabetic nephropathy; BUN, blood urea nitrogen; uPCR, urinary protein-to-creatinine ratio.

### 3.2 Urinary metabolomic profiling

Untargeted metabolomic profiling identified 148 endogenous urinary metabolites across the three study groups (**Supplementary Table 1**). PCA plots demonstrated clear separation of metabolic profiles among the groups, with quality control samples forming tight clusters, suggesting high data quality (**Supplementary Figures 1A, B**). Hierarchical clustering further revealed distinct group-specific metabolic patterns (**Supplementary Figure 1C**).

**Figure 1:**
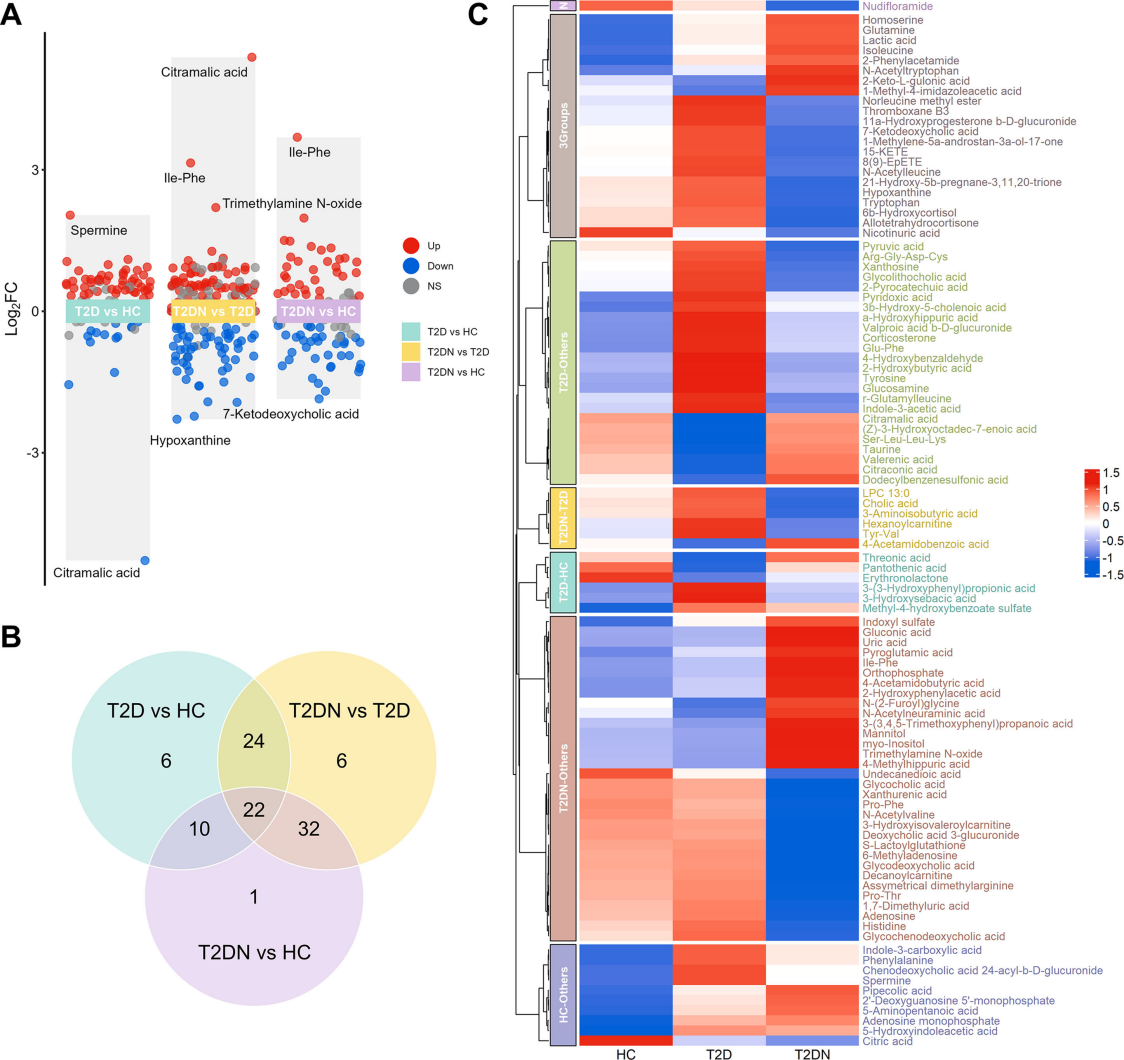
Identification and visualization of significant urinary metabolites across three groups. **(A)** Volcano plot of pairwise *post-hoc* comparisons (Dunn’s test) following a Kruskal–Wallis test on 101 differential metabolites, adjusted for age, sex, and BMI. **(B)** Venn diagram illustrating the overlap of significantly altered metabolites among the three comparisons. **(C)** Heatmap of representative metabolites categorized by the Venn diagram segmentation, showing average normalized abundance across three groups. HC, healthy controls; T2D, type 2 diabetes; T2DN, type 2 diabetic nephropathy.

Comparative analyses using the Kruskal–Wallis test (adjusted for age, sex, and BMI), followed by Dunn’s *post hoc* testing, identified 101 significantly altered metabolites (FDR < 0.05). Of these, 62 metabolites differed between T2D and HC, 84 between T2DN and T2D, and 65 between T2DN and HC (**Figure 1A**). Venn diagram analysis revealed that 30 metabolites were commonly dysregulated across all comparisons (**Figure 1B**). Heatmap visualization further confirmed distinct metabolic signatures, with widespread and consistent dysregulation particularly evident in patients with T2DN (**Figure 1C**). Collectively, these results indicate that urine metabolomic profiles reflect progressive metabolic remodeling from normoglycemia to diabetes and, ultimately, nephropathy.

### 3.3 Metabolic network and pathway analysis

**Figure 2:**
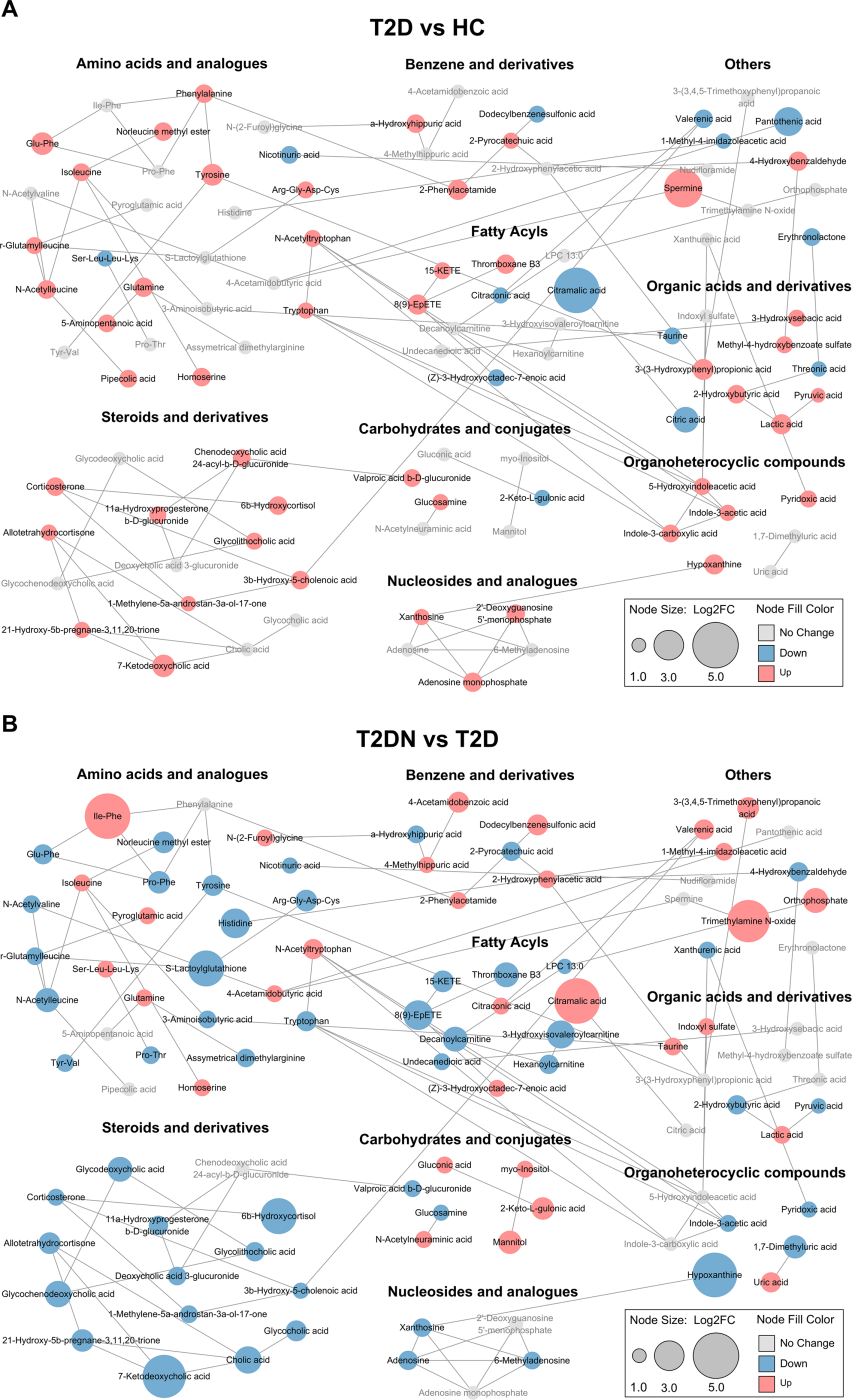
Metabolic network analysis of differentially abundant metabolites. **(A)** Comparison between patients with T2D and HC. **(B)** Comparison between patients with T2DN and T2D. Each node represents a metabolite, with node size indicating the absolute log2 fold change. Edges represent biochemical reactions and chemical similarity connections generated by MetaMapp. FC, fold change.

To elucidate the biochemical landscape of metabolic alterations, network and pathway analyses were conducted based on the 101 significantly dysregulated metabolites. MetaMapp-based metabolic network analysis revealed stage-specific shifts in metabolite classes. In T2D relative to HC, elevated levels of amino acids, peptides, organic acids, and carbohydrate-related metabolites indicated enhanced metabolic activity at the early stage of diabetes (**Figure 2A**). Conversely, the transition from T2D to T2DN exhibited broader and more complex metabolic changes. Gradual increases in metabolites such as Ile–Phe and isoleucine from HC, through T2D, to T2DN suggest progressive metabolic dysregulation along the DKD disease continuum. Concurrently, marked reductions in steroid derivatives, fatty acyls, and nucleotide-related compounds in T2DN indicated lipid metabolism and hormonal pathway suppression during nephropathy progression (**Figure 2B**).

**Figure 3:**
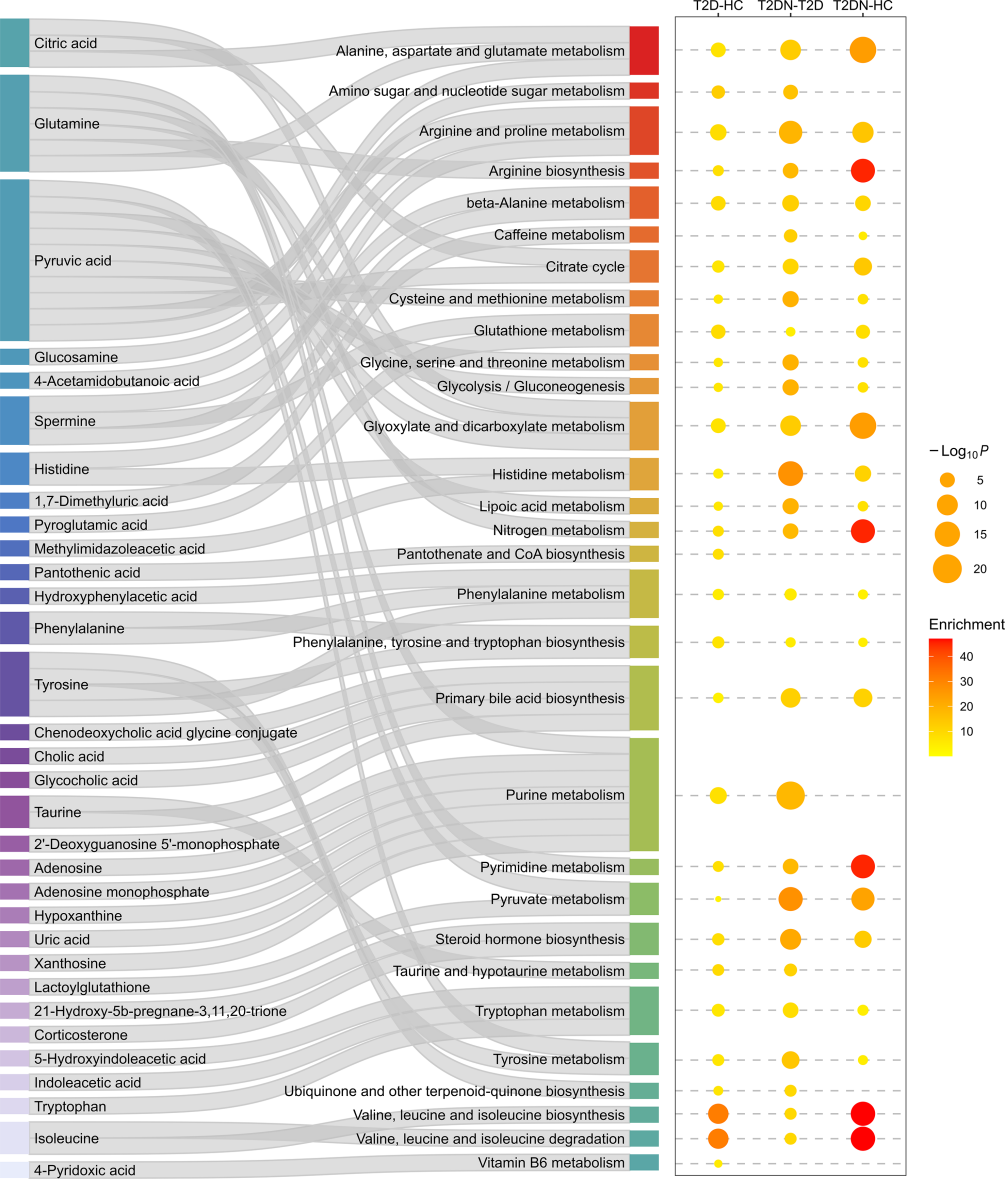
Pathway enrichment analysis of differentially abundant metabolites. Sankey plot (left) showing the mapping of altered metabolites to metabolic pathways. Bubble plot (right) displaying pathway enrichment significance and enrichment scores across three pairwise comparisons.

Pathway enrichment analysis offered further insight into the biological processes involved (**Figure 3**). Compared with HC, both T2D and T2DN samples showed significant enrichment in amino acid metabolism pathways, including arginine biosynthesis, along with glutathione, alanine, aspartate, and glutamate metabolism. Notably, T2DN exhibited unique enrichment in nitrogen metabolism, steroid hormone biosynthesis, and degradation pathways of branched-chain amino acids (BCAAs), reflecting more profound metabolic disruption associated with renal function decline. Overall, the network and pathway analyses underscore the dynamic and stage-specific metabolic remodeling that occurs during the progression from T2D to T2DN.

### 3.4 Identification of prognostic biomarkers in T2DN

**Figure 4:**
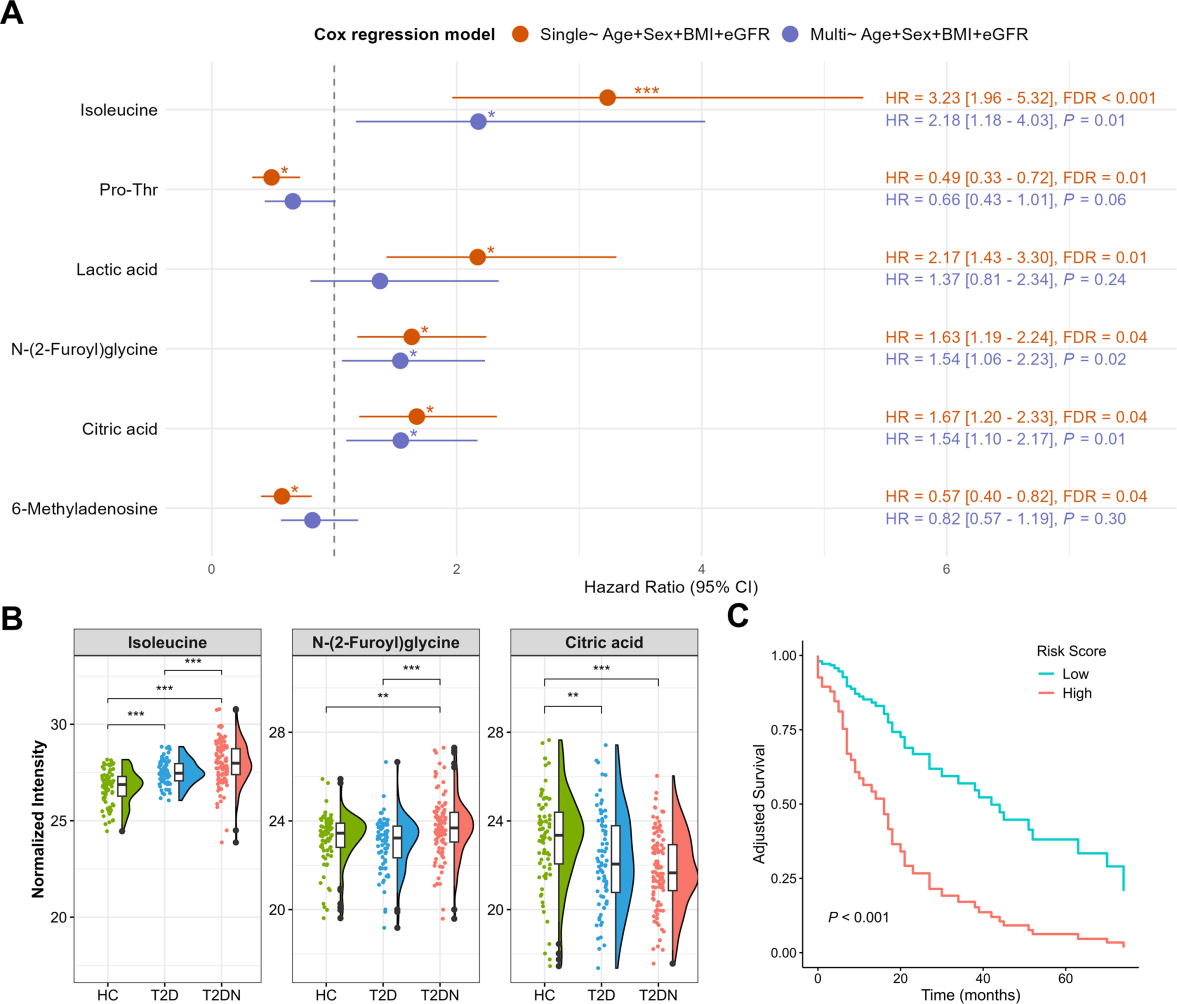
Prognostic ability of selected urinary metabolites for progression of DKD. **(A)** Forest plot of the urinary metabolites in different Cox regression models adjusted for covariates. **(B)** Group-wise distribution of the selected metabolite intensities across three groups. *FDR<0.05, **FDR<0.01, ***FDR<0.001. **(C)** Kaplan–Meier survival curves among patients with T2DN based on a composite risk score derived from three selected metabolites, adjusted for age, sex, BMI, and eGFR.

To identify urinary biomarkers predictive of DKD progression in patients with T2DN, covariate-adjusted Cox proportional hazards models were applied to the 101 metabolites identified in the cross-sectional analyses, adjusting for age, sex, BMI, and eGFR. Given the 50 observed progression events, multiplicity was controlled using the FDR procedure across the 101 metabolite-specific Cox analyses. Six metabolites met FDR < 0.05 and were considered candidate prognostic markers (**Figure 4A**). These six metabolites were subsequently entered into a multivariable Cox model with the same covariate adjustments. Three metabolites remained independently associated with renal progression: isoleucine (HR = 2.18, 95% CI: 1.18–4.03, *P* = 0.01), citric acid (HR = 1.54, 95% CI: 1.10–2.17, *P* = 0.01), and N-(2-Furoyl)glycine (HR = 1.54, 95% CI: 1.06–2.23, *P* = 0.02). Their expression patterns were further examined across the HC, T2D, and T2DN groups (**Figure 4B**).

Kaplan–Meier survival analyses demonstrated that elevated urinary levels of each biomarker were associated with significantly poorer renal survival. A combined risk score derived from the three metabolites revealed significant differences in renal survival between groups (**Figure 4C**). Higher isoleucine, citric acid, and N-(2-Furoyl)glycine levels were each associated with worse renal outcomes (**Supplementary Figure 2**). Among them, isoleucine showed the strongest prognostic value, with the most pronounced separation in survival curves (*P* < 0.0001). Collectively, these results highlight the potential utility of urinary metabolites, particularly isoleucine, as prognostic biomarkers of renal outcome in patients with T2DN.

Schoenfeld residual tests showed no violation of proportional hazards for the three selected metabolites (all *P* > 0.5), and the global test was not statistically significant (*P* = 0.065) (**Supplementary Table 2**). Although baseline eGFR showed evidence of non-proportionality, sensitivity analysis allowing a time-varying effect did not materially change the estimates (**Supplementary Table 3**). To evaluate model stability and potential overfitting, bootstrap internal validation (1000 resamples) was performed. The apparent C-index of the multivariable model was 0.823, and the optimism-corrected C-index was 0.797 (**Supplementary Table 4**), indicating limited overfitting.

### 3.5 Associations between urinary metabolites and renal pathology

**Figure 5:**
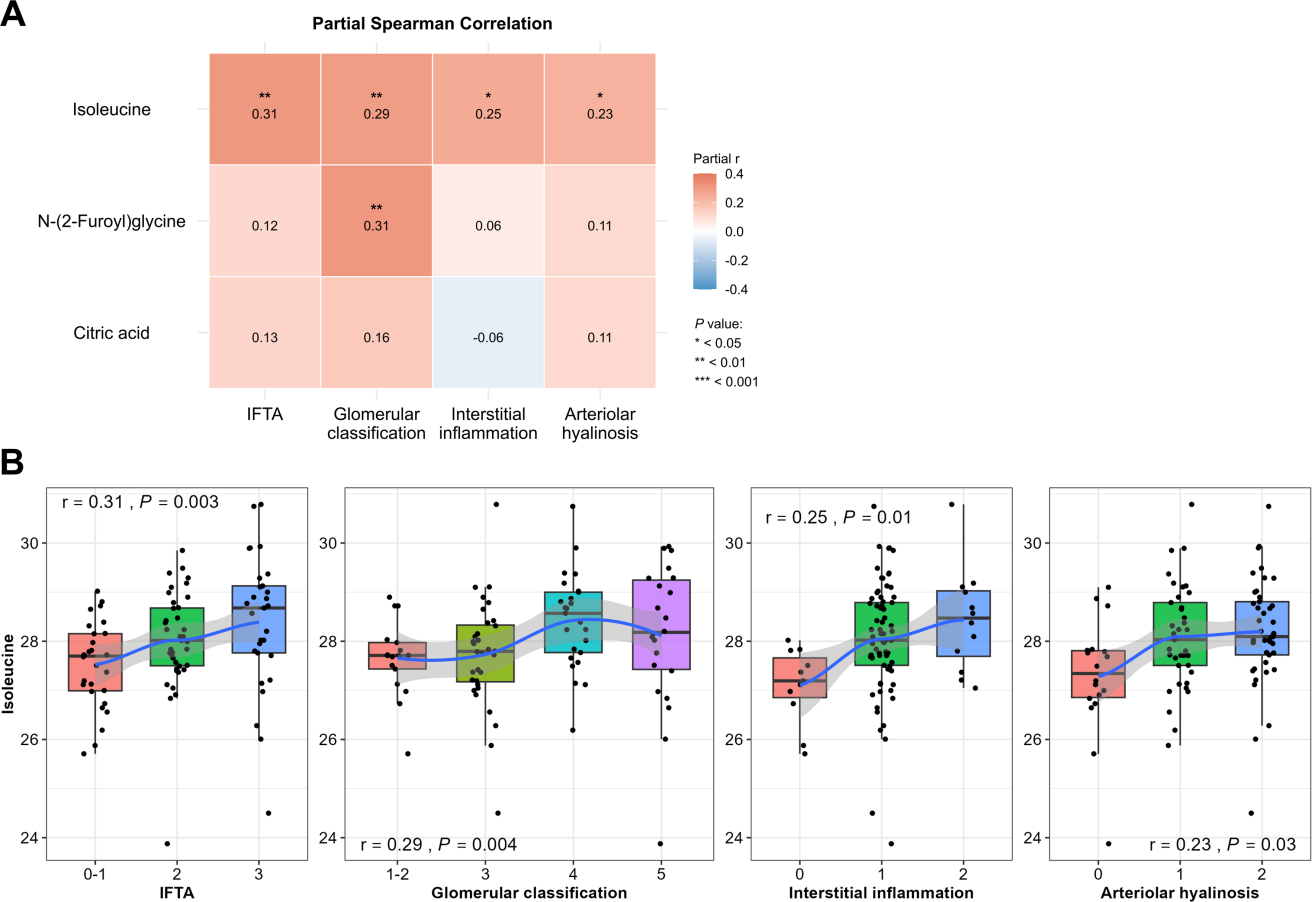
Associations between urinary metabolites and renal histological scores in patients with T2DN. **(A)** Partial Spearman correlation matrix adjusted for age, sex, and BMI. **(B)** Box plots of qualitative urinary isoleucine levels by untargeted metabolomics across different pathology scores with LOESS trend lines. *P<0.05, **P<0.01, ***P<0.001.

To explore the association between urinary metabolites and renal pathology, partial Spearman correlation was conducted after adjusting for confounders. Among the metabolites identified in Cox regression, isoleucine and N-(2-Furoyl)glycine showed significant associations with at least one pathological index, including IFTA, glomerular classification, interstitial inflammation, and arteriolar hyalinosis (**Figure 5A**). Notably, urinary isoleucine exhibited positive correlations with all four indices, most strongly with IFTA (r = 0.31, *P* = 0.0026), suggesting a potential link to tissue-level damage. Visualization using boxplots and LOESS regression demonstrated a stepwise increase in urinary isoleucine concentrations with worsening severity of each pathological score (**Figure 5B**). These findings suggest that elevated urinary isoleucine levels not only predict renal outcomes but may also reflect underlying histological damage.

### 3.6 Quantified isoleucine in different sources and DKD progression

**Figure 6:**
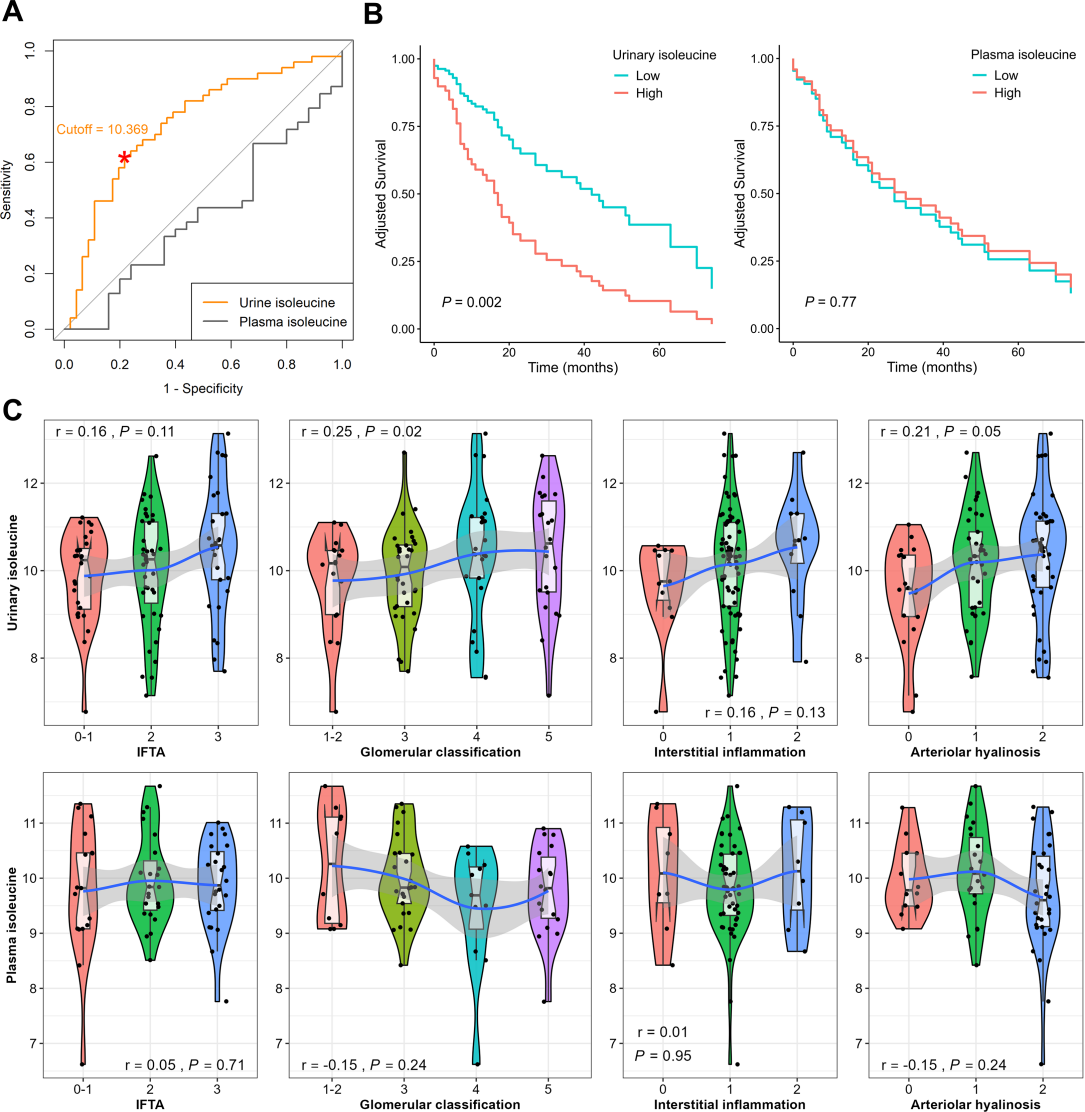
Prognostic relevance of urinary and plasma isoleucine levels in patients with T2DN. **(A)** ROC curves based on log2-transformed urinary and plasma isoleucine concentrations for predicting renal progression. A valid cutoff was identified using the Youden index, whereas no significant cutoff was found for plasma isoleucine. **(B)** Kaplan–Meier survival curves comparing patients with high versus low urinary or plasma isoleucine levels, adjusted for age, sex, BMI, and eGFR. **(C)** Violin plots showing the distribution of urinary and plasma isoleucine levels across renal histological scores, with LOESS regression lines indicating trends.

To further validate the prognostic significance of isoleucine, absolute quantification was performed in urine (*n* = 96) and available plasma (*n* = 64) samples. ROC analysis was conducted using log2-transformed urinary isoleucine concentrations. The optimal cutoff determined by the Youden index corresponded to 10.09 μmol/L in the original concentration scale (**Figure 6A**). Kaplan–Meier survival analysis confirmed that patients with urinary isoleucine levels above this cutoff had significantly poorer renal survival than those below the cutoff (**Figure 6B**). In contrast, plasma isoleucine levels showed no significant association with renal outcomes (*P* = 0.77), suggesting that urinary isoleucine better reflects kidney-specific metabolic alterations relevant to disease progression. Correlation analyses further supported that urinary, but not plasma, isoleucine levels were significantly associated with key pathological features, including glomerular classification and arteriolar hyalinosis (**Figure 6C**). Although the association between urinary isoleucine and interstitial inflammation was not significant, LOESS regression indicated consistent trends of increasing urinary isoleucine with worsening pathology. These findings emphasize the clinical relevance of urinary isoleucine as a kidney-specific biomarker reflecting both disease progression and structural renal damage.

Given the extended inclusion period of this study, we performed additional sensitivity analyses to evaluate whether storage duration influenced the observed association between urinary isoleucine and DKD progression. Storage time differed statistically across study groups but showed substantial overlap (**Supplementary Figure 3A**). In the overall cohort, storage duration exhibited only a weak correlation with urinary isoleucine (Spearman R = 0.16, *P* = 0.0096), explaining approximately 2–3% of variance. Importantly, within the T2DN cohort used for prognostic analyses, no significant correlation was observed (R = 0.097, *P* = 0.35) (**Supplementary Figures 3B, C**). Furthermore, when storage duration was included as an additional covariate in multivariable Cox models, urinary isoleucine remained significantly associated with DKD progression, whereas storage duration itself was not statistically significant (**Supplementary Table 5**). These findings indicate that the primary prognostic association was robust to potential storage-related variability.

### 3.7 Exploratory Mendelian randomization analysis

As an exploratory mechanistic complement to the clinical and quantitative findings, we conducted a one-sample MR analysis to evaluate the association between genetically predicted urinary isoleucine levels and DKD progression in 64 patients with T2DN who had available GWAS data. After stringent quality control and population stratification adjustment, thirteen SNPs significantly associated with urinary isoleucine (*P* < 0.0001, F-statistic > 20) were selected as instrumental variables (**Supplementary Table 6**). Two IV-based models consistently suggested a potential causal relationship: the TSPS model (COR = 2.06, 95% CI: 1.12–3.81, *P* = 0.021) and the MSMM (CRR = 2.26, 95% CI: 2.15–2.39, *P* < 0.001) (**Supplementary Table 7**). Given the modest sample size and exploratory design, these findings should be interpreted cautiously. The MR analysis provides preliminary genetic support for the observed association but does not establish causality, and independent validation in larger cohorts is required.

## 4 Discussion

In this study, we systematically profiled urinary metabolomic changes across HC, T2D, and biopsy-proven T2DN groups. Of the 101 significantly altered metabolites, urinary isoleucine emerged as a promising non-invasive biomarker for predicting DKD progression in the T2DN cohort. Unlike its plasma counterpart, urinary isoleucine consistently correlated with adverse renal outcomes, greater histopathological damage, and unfavorable metabolic profiles. These findings suggest that urinary metabolites, particularly isoleucine, may more accurately reflect localized renal microenvironmental changes than systemic plasma markers, highlighting their potential for early risk stratification and personalized disease monitoring in DKD.

Our untargeted metabolomic analysis revealed extensive, stage-specific metabolic remodeling across groups. Consistent with previous studies, individuals with T2D showed increased urinary excretion of amino acids, organic acids, and carbohydrate-related intermediates, reflecting a state of enhanced metabolic activity or overflow during early disease stages (21). With progression to overt nephropathy, patients with T2DN demonstrated broader dysregulation, including marked reductions in steroid derivatives, fatty acyls, and nucleotide-related metabolites, suggesting a shift from metabolic activation in preclinical diabetes to functional impairment and metabolic suppression in advanced CKD (17, 22, 23). Pathway enrichment analysis further revealed significant disturbances in nitrogen and amino acid metabolism, particularly in the T2DN group, highlighting the increasing metabolic burden associated with renal dysfunction (24). This global remodeling provides a mechanistic context for the selective elevation of isoleucine and supports the hypothesis that specific metabolites may act as both biomarkers and potential mediators of progressive kidney injury.

A prominent metabolic signature in T2DN was BCAA metabolism enrichment. BCAAs, including leucine, isoleucine, and valine, are essential amino acids that not only support protein synthesis but also contribute to insulin resistance and renal dysfunction through dysregulated catabolism and tissue-specific metabolic signaling (25, 26). Notably, only isoleucine exhibited a robust and reproducible association with DKD progression in our data. This specificity may stem from its dual catabolic fate (generating both glucogenic and ketogenic intermediates), which under pathological conditions promotes mitochondrial stress in proximal tubules (22). Unlike leucine, which primarily activates mammalian target of rapamycin complex 1 (mTORC1) via transient Sestrin2–GATOR2 signaling in muscle and liver, isoleucine may contribute to sustained mTORC1 activation in renal proximal tubular cells, potentially through alternative nutrient-sensing pathways such as CASTOR1 or Rag GTPases (27, 28). Such prolonged activation could promote downstream stress responses or fibrotic signaling, ultimately contributing to tubular injury. In addition, a large multicohort study identified adenine as a metabolite that stimulates mTOR signaling in tubular cells and promotes kidney fibrosis (29). Specifically, the urinary adenine-to-creatinine ratio predicted DKD progression and mortality, even among patients without macroalbuminuria. Although adenine was not detected in our platform, these findings underscore the broader relevance of metabolite-driven mTOR activation in DKD pathogenesis.

Critically, proximal tubules express key transporters, including apical sodium-dependent neutral amino acid transporter B^0^AT1 (*SLC6A19*) and the basolateral heterodimeric LAT2–4F2hc complex (*SLC7A8*–*SLC3A2*) (30). Although direct evidence regarding their regulation under diabetic stress remains limited, experimental models have demonstrated that B^0^AT1 deficiency attenuates nephropathy progression, and LAT2 contributes to mTORC1 pathway activation and glomerular injury, implying these transporters may be susceptible to dysfunction under metabolic or inflammatory stress (31, 32). Although B^0^AT1 exhibits slightly higher affinity for leucine than for isoleucine, both substrates compete for the same transporter. Under diabetic conditions with transporter downregulation, isoleucine reabsorption may be disproportionately impaired due to altered substrate availability or transporter saturation thresholds (33). The strong correlation between urinary isoleucine and IFTA scores further supports its role as a structural–functional integrator of early tubulointerstitial damage, suggesting that persistent isoleucine leakage contributes to profibrotic signaling cascades.

Emerging evidence suggests that the gut microbiota is crucial in systemic amino acid homeostasis, contributing to aminoaciduria through the gut–kidney metabolic axis (34). In T2D, bacterial taxa such as *Prevotella copri* and *Bacteroides vulgatus* are enriched and exhibit enhanced *de novo* BCAA biosynthesis via microbial BCAA aminotransferase and branched-chain keto acid dehydrogenase gene clusters (35, 36). This overproduction elevates circulating isoleucine levels, which may exceed the reabsorptive capacity of damaged proximal tubules in T2DN. In progressive tubular injury, characterized by oxidative stress, mitochondrial dysfunction, and ATP depletion, neutral amino acid transporters such as B^0^AT1 may become dysfunctional, further impairing isoleucine reabsorption and contributing to its persistent urinary loss (37–39). In addition, microbial-derived BCAAs may exacerbate systemic insulin resistance and inflammation, thereby amplifying renal metabolic stress (40). Together, these processes may create a vicious cycle where gut-derived BCAA overload promotes tubular dysfunction and accelerates kidney injury. Although direct causal evidence in DKD is limited, the gut–kidney axis is increasingly recognized as a key modulator of amino acid metabolism and early tubular damage (41). Further investigation is warranted to determine whether targeting microbial BCAA production could offer therapeutic benefit in DKD.

Aminoaciduria has long been recognized as a feature of proximal tubular dysfunction, supporting the rationale for urinary isoleucine as an early and accessible tubular injury biomarker (12, 42). Unlike conventional measures such as serum creatinine or eGFR, which primarily reflect glomerular filtration, urinary isoleucine may capture upstream tubular damage. This concept is supported by established tubular markers such as urinary KIM-1, which correlates with eGFR decline and albuminuria across multiple cohorts (43). Moreover, the association of urinary isoleucine with histopathological lesions highlights its potential to bridge metabolic perturbations and structural pathology. Future studies should evaluate whether urinary isoleucine adds prognostic value beyond UACR, NGAL, or KIM-1 using risk reclassification metrics such as net reclassification improvement and integrated discrimination improvement (44).

In parallel, these findings highlight the therapeutic potential of targeting BCAA-related metabolic pathways in DKD. Preclinical studies have demonstrated that dietary BCAA restriction can improve metabolic health and attenuate renal injury in diabetic models (26, 45, 46). Similarly, gut microbiota modulation through probiotics, prebiotics, or short-chain fatty acid supplementation reportedly reduces kidney injury and systemic metabolic stress (47–49). However, systemic BCAA depletion poses risks, including sarcopenia or metabolic imbalance, particularly in patients with CKD who have reduced muscle mass (50). Likewise, pharmacologic mTORC1 inhibitors may exacerbate proteinuria or impair tissue repair, underscoring the need for kidney-specific mTORC1 modulation strategies (51–53). Finally, analytical challenges, including assay standardization, reference range establishment, and individual variability minimization, remain key hurdles for the clinical translation of amino acid–based biomarkers.

This study has some limitations. First, the modest sample size and single-center design, comprising exclusively Korean participants, may limit the generalizability of our findings to other ethnicities and underrepresented populations. Validation in larger, multi-center cohorts encompassing diverse ancestries, clinical backgrounds, and healthcare settings is warranted to ensure broader applicability. Second, the extended inclusion period (2011–2022) may introduce potential storage- or time-related variability. Although standardized processing, −80 °C storage, batch randomization, and sensitivity analyses were applied, residual temporal effects cannot be entirely excluded. Third, early or subclinical DKD in the T2D control group cannot be entirely excluded based on clinical criteria alone, and some degree of misclassification may persist. Fourth, despite multivariable adjustment, residual confounding may remain. In particular, diabetes duration, glycemic control, blood pressure, and medication use were not comprehensively adjusted for and may influence metabolomic profiles and renal outcomes. Finally, although our findings suggest mechanistic links between isoleucine and renal injury, these remain hypothetical. Experimental validation using *in vitro* assays on tubular epithelial cells and *in vivo* diabetic nephropathy models is needed to clarify the biological relevance and therapeutic potential of isoleucine-related pathways.

## 5 Conclusion

In conclusion, we identified urinary isoleucine as a robust and mechanistically plausible biomarker for DKD progression in patients with T2DN. By integrating untargeted and targeted metabolomics with histopathological and clinical outcome data, we demonstrate the diagnostic, prognostic, and potential mechanistic relevance of a single metabolite. These findings support a growing paradigm that emphasizes urine-based metabolic profiling as a non-invasive strategy for detecting subclinical kidney injury and guiding interventions targeting amino acid metabolism, proximal tubular health, and systemic metabolic homeostasis in DKD.

## Data Availability

The datasets presented in this study can be found in online repositories. The names of the repository/repositories and accession number(s) can be found below: https://kbds.re.kr/, KAP241555.
