# Supplementary material for: Urinary metabolomics identifies isoleucine as a prognostic biomarker for progression of diabetic kidney disease
Source: Front Endocrinol (Lausanne). 2026 Mar 23;17:1787886. doi: 10.3389/fendo.2026.1787886 (PMC13050695; doi:10.3389/fendo.2026.1787886)
Supplement: Supplementary file 1 [file DataSheet1.docx]

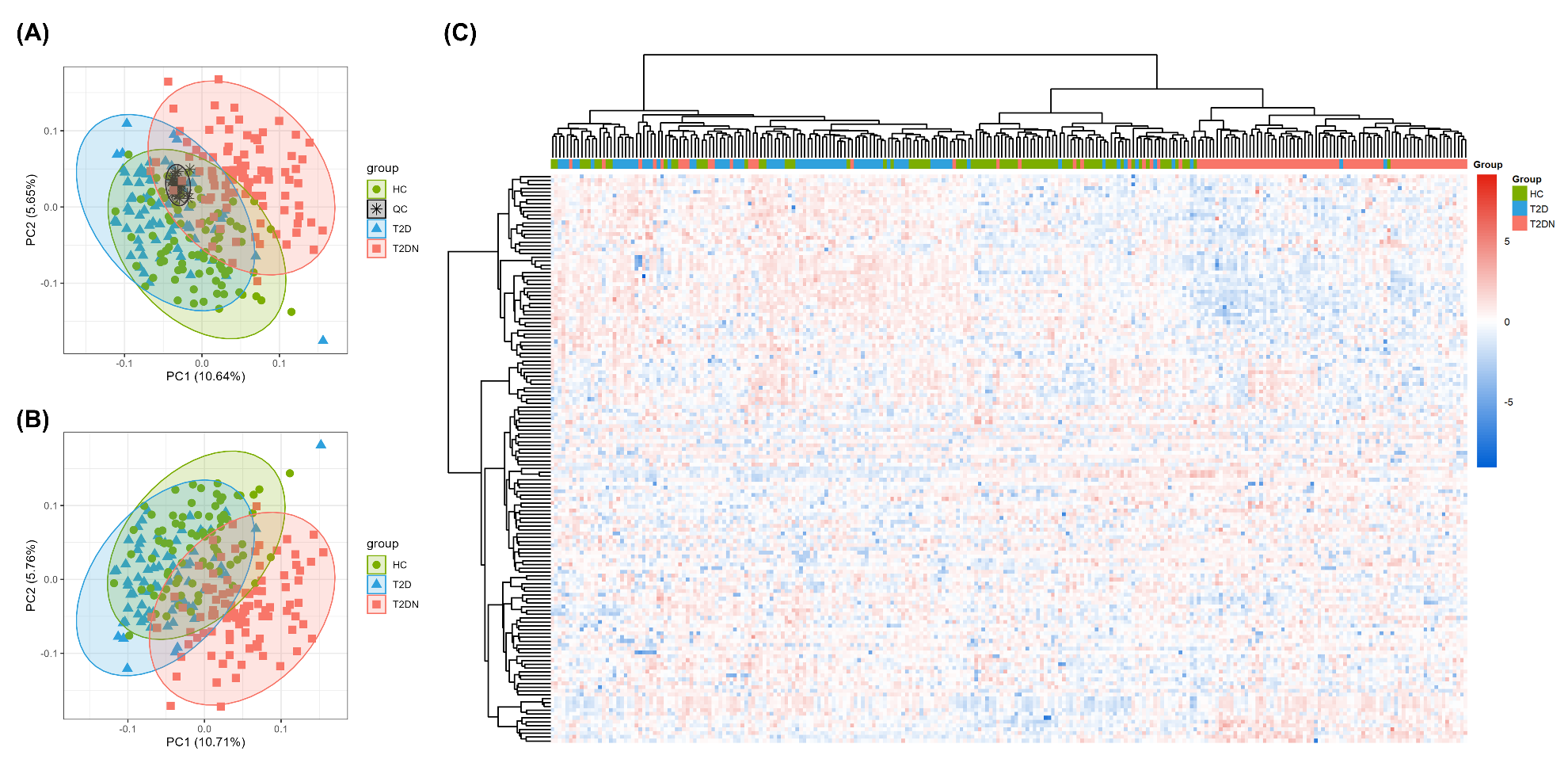


**Supplementary Figure S1.** Multivariate analysis of 148 endogenous urinary metabolites. (A) Principal component analysis (PCA) plot including quality control samples. (B) PCA plot excluding QC samples. (C) Hierarchical clustering heatmap of metabolite abundance across study groups. HC: healthy controls; QC: quality control; T2D: type 2 diabetes without nephropathy; T2DN: type 2 diabetic nephropathy.


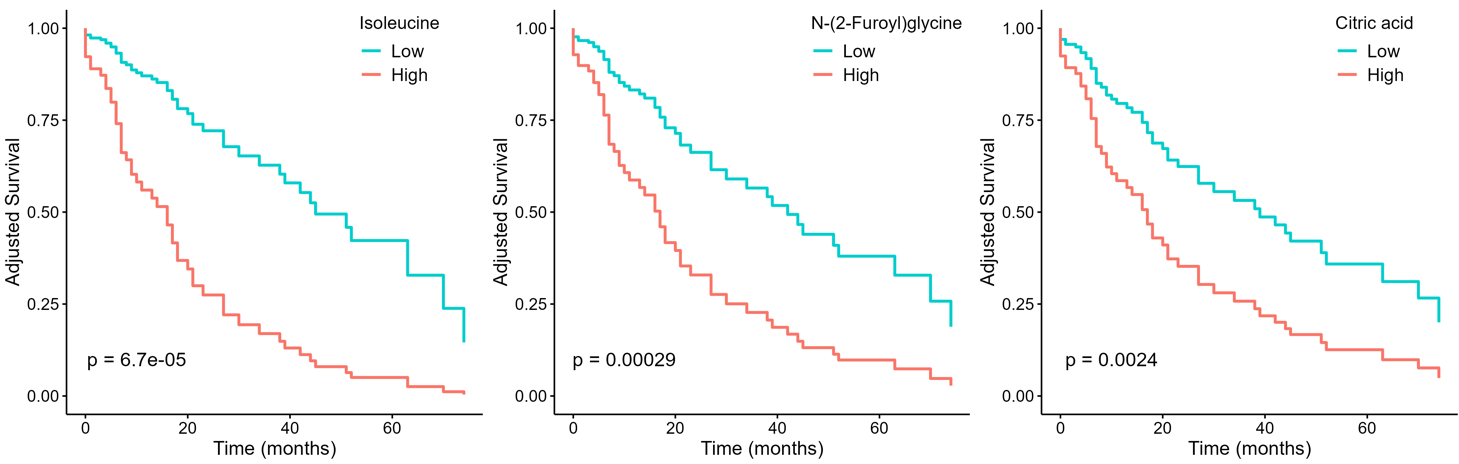


**Supplementary Figure S2.** Kaplan–Meier survival curves among patients with type 2 diabetic nephropathy based on three individual metabolites. Kaplan–Meier survival curves among patients with type 2 diabetic nephropathy (T2DN) based on three individual metabolites, adjusted for age, sex, BMI, and eGFR.


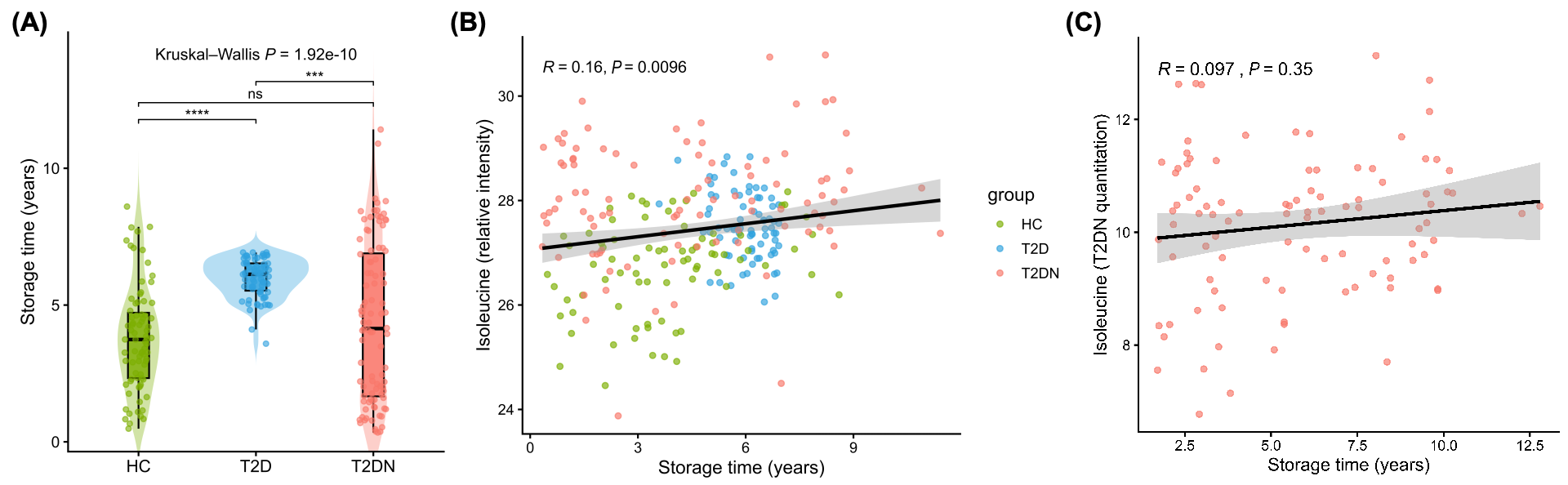


**Supplementary Figure S3.** Evaluation of storage duration and its potential influence on urinary isoleucine levels. (A) Storage time distribution across HC, T2D, and T2DN groups. (B) Weak correlation between storage time and urinary isoleucine in the overall cohort. (C) No significant correlation within the T2DN cohort.

Supplementary Table S1. Endogenous metabolites identified in urine samples through untargeted metabolomics

| Compound | *m/z* | RT (s) | Adduct | *m/z* error (ppm) | MS/MS Similarity | Total score | MSI Level |
| --- | --- | --- | --- | --- | --- | --- | --- |
| 2-Hydroxybutyric acid | 103.0399 | 80.96 | (M-H)- | 0.57 | 0.74 | 0.84 | 2 |
| Adenosine | 268.1034 | 38.64 | (M+H)+ | 0.09 | 0.68 | 0.75 | 2 |
| Carnitine | 162.1122 | 34.97 | (M+H)+ | 1.76 | 0.54 | 0.77 | 2 |
| Pipecolic acid | 130.0861 | 37.59 | (M+H)+ | 0.01 | 0.73 | 0.83 | 2 |
| cis-Aconitic acid | 173.0089 | 93.14 | (M-H)- | 0.78 | 0.75 | 0.84 | 2 |
| Citric acid | 191.0195 | 84.89 | (M-H)- | 1.13 | 0.74 | 0.82 | 2 |
| Glycolic acid | 75.0086 | 35.93 | (M-H)- | 0.27 | 0.76 | 0.85 | 2 |
| Glycocholic acid | 464.3021 | 517.91 | (M-H)- | 2.77 | 0.71 | 0.80 | 2 |
| Tyrosine | 182.0811 | 42.30 | (M+H)+ | 0.00 | 0.60 | 0.75 | 2 |
| Phenylalanine | 166.0861 | 111.68 | (M+H)+ | 0.04 | 0.77 | 0.86 | 2 |
| Histidine | 156.0765 | 38.90 | (M+H)+ | 0.03 | 0.77 | 0.85 | 2 |
| Lysine | 147.1127 | 29.74 | (M+H)+ | 0.07 | 0.67 | 0.83 | 2 |
| Lactic acid | 89.0243 | 44.83 | (M-H)- | 0.44 | 0.71 | 0.82 | 2 |
| Indole-3-acetic acid | 176.0704 | 339.98 | (M+H)+ | 0.08 | 0.65 | 0.78 | 2 |
| Acetylcarnitine | 204.1228 | 38.12 | (M+H)+ | 0.04 | 0.70 | 0.81 | 2 |
| myo-Inositol | 179.0557 | 36.72 | (M-H)- | 0.90 | 0.64 | 0.77 | 2 |
| N-Acetylneuraminic acid | 308.0983 | 36.19 | (M-H)- | 1.43 | 0.69 | 0.84 | 2 |
| Taurine | 124.0071 | 34.62 | (M-H)- | 0.56 | 0.75 | 0.85 | 2 |
| Pyroglutamic acid | 130.0497 | 44.92 | (M+H)+ | 0.02 | 0.71 | 0.85 | 2 |
| Uric acid | 167.0206 | 46.40 | (M-H)- | 0.93 | 0.80 | 0.87 | 2 |
| Xanthosine | 283.0683 | 100.07 | (M-H)- | 1.25 | 0.71 | 0.82 | 2 |
| Allotetrahydrocortisone | 365.2328 | 482.92 | (M+H)+ | 0.41 | 0.64 | 0.78 | 2 |
| Erythronolactone | 117.0191 | 51.90 | (M-H)- | 0.66 | 0.72 | 0.83 | 2 |
| 3-Hydroxysebacic acid | 217.1076 | 355.59 | (M-H)- | 1.08 | 0.50 | 0.69 | 2 |
| 3-(3-Hydroxyphenyl)propionic acid | 165.0554 | 358.48 | (M-H)- | 0.79 | 0.71 | 0.82 | 2 |
| 7-Ketodeoxycholic acid | 405.2638 | 607.80 | (M+H)+ | 0.57 | 0.63 | 0.77 | 2 |
| 2-Pyrocatechuic acid | 153.0191 | 309.52 | (M-H)- | 0.68 | 0.63 | 0.77 | 2 |
| Citramalic acid | 147.0297 | 38.03 | (M-H)- | 0.83 | 0.73 | 0.83 | 2 |
| N-(2-Furoyl)glycine | 168.0300 | 111.07 | (M-H)- | 0.77 | 0.70 | 0.81 | 2 |
| 3-Methyladipic acid | 159.0660 | 287.53 | (M-H)- | 0.77 | 0.74 | 0.84 | 2 |
| Creatinine | 114.0657 | 283.69 | (M+H)+ | 0.05 | 0.71 | 0.82 | 2 |
| Cholic acid | 407.2800 | 573.42 | (M-H)- | 3.53 | 0.72 | 0.79 | 2 |
| Dodecanedioic acid | 229.1440 | 572.37 | (M-H)- | 1.08 | 0.53 | 0.71 | 2 |
| Gluconic acid | 195.0504 | 35.15 | (M-H)- | 0.93 | 0.67 | 0.79 | 2 |
| Citraconic acid | 129.0191 | 93.79 | (M-H)- | 0.67 | 0.71 | 0.82 | 2 |
| Glycochenodeoxycholic acid | 432.3110 | 634.25 | (M+H-H2O)+ | 0.54 | 0.63 | 0.77 | 2 |
| Glutamine | 145.0616 | 288.83 | (M-H)- | 0.71 | 0.73 | 0.83 | 2 |
| Decanoylcarnitine | 316.2484 | 547.06 | (M+H)+ | 0.24 | 0.71 | 0.82 | 2 |
| 2-Hydroxyphenylacetic acid | 151.0398 | 306.38 | (M-H)- | 0.89 | 0.72 | 0.82 | 2 |
| Indoxyl sulfate | 212.0018 | 305.07 | (M-H)- | 1.25 | 0.65 | 0.78 | 2 |
| Hippuric acid | 180.0653 | 283.43 | (M+H)+ | 0.07 | 0.79 | 0.87 | 2 |
| Kynurenic acid | 188.0350 | 243.28 | (M-H)- | 0.71 | 0.76 | 0.85 | 2 |
| Hexanoylcarnitine | 260.1853 | 327.15 | (M+H)+ | 0.29 | 0.63 | 0.77 | 2 |
| 5-Hydroxyindoleacetic acid | 192.0657 | 258.29 | (M+H)+ | 0.13 | 0.69 | 0.81 | 2 |
| Mannitol | 181.0714 | 34.88 | (M-H)- | 0.86 | 0.57 | 0.78 | 2 |
| Azelaic acid | 187.0973 | 395.65 | (M-H)- | 1.06 | 0.74 | 0.83 | 2 |
| Octanoylcarnitine | 288.2168 | 473.23 | (M+H)+ | 0.27 | 0.72 | 0.83 | 2 |
| Sebacic acid | 201.1127 | 451.15 | (M-H)- | 0.89 | 0.74 | 0.83 | 2 |
| Xanthurenic acid | 206.0446 | 220.07 | (M+H)+ | 0.35 | 0.69 | 0.81 | 2 |
| Undecanedioic acid | 215.1284 | 524.72 | (M-H)- | 1.11 | 0.66 | 0.78 | 2 |
| Suberic acid | 173.0816 | 359.78 | (M-H)- | 0.72 | 0.72 | 0.82 | 2 |
| Trimethylamine N-oxide | 76.0756 | 44.14 | (M+H)+ | 0.11 | 0.71 | 0.82 | 2 |
| Threonic acid | 135.0296 | 35.93 | (M-H)- | 0.68 | 0.65 | 0.78 | 2 |
| Tiglylglycine | 156.0663 | 217.36 | (M-H)- | 0.62 | 0.61 | 0.76 | 2 |
| 3-Succinoylpyridine | 178.0516 | 779.46 | (M-H)- | 0.68 | 0.70 | 0.81 | 2 |
| 2'-Deoxyguanosine 5'-monophosphate | 346.0541 | 200.87 | (M-H)- | 5.66 | 0.67 | 0.74 | 2 |
| S-Lactoylglutathione | 378.1017 | 587.55 | (M-H)- | 7.81 | 0.52 | 0.60 | 2 |
| 5'-Deoxy-5'-(methylthio)adenosine | 298.0968 | 223.74 | (M+H)+ | 0.21 | 0.60 | 0.76 | 2 |
| Ne,Ne,Ne-Trimethyllysine | 189.1596 | 31.83 | (M+H)+ | 0.07 | 0.69 | 0.81 | 2 |
| Orthophosphate | 96.9694 | 43.00 | (M-H)- | 0.42 | 0.77 | 0.86 | 2 |
| Glucosamine | 180.0879 | 197.29 | (M+H)+ | 3.43 | 0.59 | 0.72 | 2 |
| Assymetrical dimethylarginine | 203.1502 | 34.97 | (M+H)+ | 0.13 | 0.71 | 0.82 | 2 |
| Corticosterone | 347.2220 | 467.74 | (M+H)+ | 0.09 | 0.73 | 0.83 | 2 |
| Norleucine | 132.1017 | 45.18 | (M+H)+ | 0.01 | 0.73 | 0.83 | 2 |
| Methyl-4-hydroxybenzoate sulfate | 230.9967 | 263.44 | (M-H)- | 2.26 | 0.65 | 0.80 | 2 |
| 1,3-Dimethyluric acid | 195.0518 | 200.35 | (M-H)- | 0.98 | 0.74 | 0.84 | 2 |
| 3-Methyluric acid | 183.0511 | 78.17 | (M+H)+ | 0.06 | 0.59 | 0.74 | 2 |
| 7-Methylxanthine | 167.0561 | 72.15 | (M+H)+ | 0.11 | 0.56 | 0.72 | 2 |
| 1,9-Dimethyluric acid | 197.0667 | 176.09 | (M+H)+ | 0.42 | 0.66 | 0.78 | 2 |
| 1,3,7-Trimethyluric acid | 209.0677 | 250.35 | (M-H)- | 0.76 | 0.66 | 0.79 | 2 |
| a-Hydroxyhippuric acid | 196.0604 | 347.83 | (M+H)+ | 0.18 | 0.71 | 0.82 | 2 |
| 4-Acetamidobenzoic acid | 178.0514 | 330.46 | (M-H)- | 0.60 | 0.70 | 0.81 | 2 |
| 3,4,5-Trimethoxycinnamic acid | 239.0887 | 339.71 | (M+H)+ | 5.57 | 0.75 | 0.79 | 2 |
| Deoxycholic acid 3-glucuronide | 567.3144 | 559.54 | (M-H)- | 0.66 | 0.60 | 0.78 | 2 |
| Androsterone sulfate | 369.1738 | 510.59 | (M-H)- | 0.40 | 0.69 | 0.78 | 2 |
| 1-Methyl-4-imidazoleacetic acid | 141.0656 | 36.81 | (M+H)+ | 0.09 | 0.51 | 0.76 | 2 |
| Nicotinuric acid | 179.0462 | 232.29 | (M-H)- | 0.62 | 0.58 | 0.74 | 2 |
| Indole-3-carboxylic acid | 162.0549 | 315.11 | (M+H)+ | 0.00 | 0.67 | 0.79 | 2 |
| 5-Aminopentanoic acid | 118.0861 | 44.92 | (M+H)+ | 0.09 | 0.74 | 0.84 | 2 |
| 4-Acetamidobutyric acid | 144.0664 | 54.52 | (M-H)- | 0.77 | 0.73 | 0.83 | 2 |
| 3-Aminoisobutyric acid | 104.0704 | 36.81 | (M+H)+ | 0.01 | 0.52 | 0.70 | 2 |
| 6-Methyladenosine | 282.1194 | 37.85 | (M+H)+ | 0.27 | 0.60 | 0.75 | 2 |
| Nudifloramide | 153.0656 | 45.18 | (M+H)+ | 0.07 | 0.76 | 0.85 | 2 |
| N,N-Dimethylguanosine | 312.1302 | 197.29 | (M+H)+ | 0.34 | 0.71 | 0.82 | 2 |
| Thromboxane B3 | 367.2124 | 527.86 | (M-H)- | 2.28 | 0.71 | 0.81 | 2 |
| o-Tyrosine | 180.0663 | 305.59 | (M-H)- | 0.63 | 0.54 | 0.71 | 2 |
| 21-Hydroxy-5b-pregnane-3,11,20-trione | 347.2220 | 483.18 | (M+H)+ | 0.55 | 0.70 | 0.81 | 2 |
| 2-Phenylacetamide | 136.0755 | 288.40 | (M+H)+ | 0.19 | 0.66 | 0.79 | 2 |
| 1-Methylxanthine | 165.0416 | 107.93 | (M-H)- | 2.20 | 0.76 | 0.86 | 2 |
| 1,7-Dimethyluric acid | 197.0666 | 199.39 | (M+H)+ | 0.19 | 0.68 | 0.80 | 2 |
| r-Glutamylleucine | 259.1292 | 213.17 | (M-H)- | 1.27 | 0.55 | 0.72 | 2 |
| 4-Hydroxybenzaldehyde | 121.0293 | 229.41 | (M-H)- | 0.50 | 0.78 | 0.86 | 2 |
| Homovanillic acid sulfate | 261.0070 | 242.50 | (M-H)- | 1.40 | 0.70 | 0.81 | 2 |
| N-Acetylleucine | 172.0976 | 321.82 | (M-H)- | 0.68 | 0.68 | 0.80 | 2 |
| 1-Methylhypoxanthine | 151.0613 | 52.25 | (M+H)+ | 0.02 | 0.52 | 0.72 | 2 |
| 4-Methylhippuric acid | 192.0663 | 316.85 | (M-H)- | 0.80 | 0.55 | 0.72 | 2 |
| N-Acetyltryptophan | 245.0929 | 382.30 | (M-H)- | 1.27 | 0.54 | 0.71 | 2 |
| Octadecylamine | 270.3142 | 652.31 | (M+H)+ | 0.55 | 0.70 | 0.81 | 2 |
| Valerenic acid | 233.1539 | 765.06 | (M-H)- | 1.24 | 0.61 | 0.76 | 2 |
| 3-(3,4,5-Trimethoxyphenyl)propanoic acid | 223.0962 | 570.10 | (M+H-H2O)+ | 0.40 | 0.64 | 0.77 | 2 |
| Tryptophan | 205.0968 | 211.43 | (M+H)+ | 0.07 | 0.76 | 0.85 | 2 |
| Cyclo(Leu-Pro) | 211.1439 | 332.12 | (M+H)+ | 0.38 | 0.67 | 0.79 | 2 |
| 6b-Hydroxycortisol | 379.2118 | 420.09 | (M+H)+ | 0.76 | 0.67 | 0.79 | 2 |
| CMPF | 239.0920 | 569.75 | (M-H)- | 1.30 | 0.62 | 0.76 | 2 |
| 16a-hydroxy DHEA 3-sulfate | 383.1532 | 453.25 | (M-H)- | 4.00 | 0.67 | 0.80 | 2 |
| Arg-Gly-Asp | 345.1551 | 460.84 | (M-H)- | 4.26 | 0.65 | 0.75 | 2 |
| Arg-Gly-Asp-Cys | 448.1616 | 433.61 | (M-H)- | 3.57 | 0.56 | 0.70 | 2 |
| His-His | 293.1379 | 395.48 | (M+H)+ | 7.96 | 0.63 | 0.66 | 2 |
| Ser-Leu-Leu-Lys | 460.3125 | 324.53 | (M+H)+ | 2.13 | 0.59 | 0.74 | 2 |
| (Z)-3-Hydroxyoctadec-7-enoic acid | 297.2433 | 821.35 | (M-H)- | 1.51 | 0.65 | 0.78 | 2 |
| 11-Dehydrothromboxane B3 | 365.1965 | 444.35 | (M-H)- | 1.97 | 0.73 | 0.82 | 2 |
| 1-Methylene-5a-androstan-3a-ol-17-one | 349.2377 | 461.19 | (M+HCOO+2H)+ | 0.27 | 0.66 | 0.79 | 2 |
| 3-Hydroxyisovaleroylcarnitine | 262.1649 | 83.15 | (M+H)+ | 0.30 | 0.73 | 0.83 | 2 |
| Glycolithocholic acid | 416.3163 | 700.22 | (M+H-H2O)+ | 0.39 | 0.72 | 0.83 | 2 |
| N6-Threonylcarbamoyladenosine | 413.1424 | 240.75 | (M+H)+ | 0.54 | 0.73 | 0.83 | 2 |
| Pantothenic acid | 220.1179 | 152.26 | (M+H)+ | 0.46 | 0.77 | 0.86 | 2 |
| Pro-Phe | 263.1390 | 276.36 | (M+H)+ | 0.69 | 0.71 | 0.82 | 2 |
| Prostaglandin K2 | 349.2019 | 545.40 | (M-H)- | 2.15 | 0.71 | 0.81 | 2 |
| Pro-Thr | 217.1180 | 77.12 | (M+H)+ | 0.04 | 0.56 | 0.72 | 2 |
| Thr-Phe | 267.1316 | 326.62 | (M+H)+ | 5.86 | 0.69 | 0.74 | 2 |
| Tyr-Val | 281.1470 | 388.67 | (M+H)+ | 5.53 | 0.70 | 0.76 | 2 |
| Valproic acid b-D-glucuronide | 319.1396 | 487.28 | (M-H)- | 1.91 | 0.81 | 0.87 | 2 |
| Pyridoxic acid | 182.0456 | 69.18 | (M-H)- | 2.23 | 0.69 | 0.79 | 2 |
| Adenosine monophosphate | 348.0693 | 179.49 | (M+H)+ | 2.88 | 0.59 | 0.73 | 2 |
| Hypoxanthine | 137.0457 | 44.66 | (M+H)+ | 0.01 | 0.74 | 0.84 | 2 |
| Isocitric acid | 191.0192 | 44.83 | (M-H)- | 0.91 | 0.78 | 0.86 | 2 |
| Pyruvic acid | 87.0086 | 39.60 | (M-H)- | 0.44 | 0.61 | 0.76 | 2 |
| Vanillylmandelic acid | 197.0453 | 78.34 | (M-H)- | 0.87 | 0.62 | 0.76 | 2 |
| 3b-Hydroxy-5-cholenoic acid | 357.2790 | 605.45 | (M+H)+ | 4.18 | 0.57 | 0.66 | 2 |
| Glycodeoxycholic acid | 414.3007 | 622.99 | (M+H-2H2O)+ | 0.82 | 0.74 | 0.83 | 2 |
| Homoserine | 84.0442 | 288.66 | (M+H-2H2O)+ | 0.07 | 0.65 | 0.78 | 2 |
| Methylglutaric acid | 145.0503 | 213.96 | (M-H)- | 0.57 | 0.68 | 0.80 | 2 |
| Spermine | 203.2228 | 25.81 | (M+H)+ | 0.21 | 0.72 | 0.82 | 2 |
| Phenylacetyl-L-glutamine | 265.1188 | 288.40 | (M+H)+ | 0.40 | 0.75 | 0.84 | 2 |
| 15-KETE | 301.2162 | 556.23 | (M+H-H2O)+ | 0.38 | 0.64 | 0.77 | 2 |
| 2-Keto-L-gulonic acid | 193.0351 | 35.15 | (M-H)- | 0.95 | 0.64 | 0.78 | 2 |
| N-Acetylvaline | 158.0820 | 237.52 | (M-H)- | 0.45 | 0.51 | 0.69 | 2 |
| Dodecylbenzenesulfonic acid | 325.1836 | 647.25 | (M-H)- | 1.47 | 0.70 | 0.81 | 2 |
| Ile-Phe | 279.1698 | 325.84 | (M+H)+ | 0.43 | 0.57 | 0.73 | 2 |
| 11a-Hydroxyprogesterone b-D-glucuronide | 507.2585 | 483.71 | (M+H)+ | 0.01 | 0.55 | 0.72 | 2 |
| 3a,7a,12a-Trihydroxy-5a-cholan-24-oic acid | 407.2800 | 700.39 | (M-H)- | 2.05 | 0.69 | 0.80 | 2 |
| 8(9)-EpETE | 365.2321 | 457.53 | (M+HCOO+2H)+ | 0.53 | 0.52 | 0.70 | 2 |
| Benzyl-L-glutamine methyl ester | 251.1362 | 71.89 | (M+H)+ | 5.74 | 0.58 | 0.68 | 2 |
| Chenodeoxycholic acid 24-acyl-b-D-glucuronide | 567.3168 | 574.20 | (M-H)- | 1.11 | 0.53 | 0.70 | 2 |
| Glu-Phe | 295.1291 | 254.37 | (M+H)+ | 0.47 | 0.76 | 0.85 | 2 |
| LPC 13:0 | 454.2930 | 622.99 | (M+H)+ | 0.36 | 0.65 | 0.78 | 2 |
| Norleucine methyl ester | 128.1068 | 322.17 | (M+H-H2O)+ | 0.14 | 0.54 | 0.71 | 2 |
| Pro-Leu | 229.1548 | 74.77 | (M+H)+ | 0.10 | 0.56 | 0.72 | 2 |

**Supplementary Table S2.** Assessment of proportional hazards assumption using Schoenfeld residual tests

| Variable | χ² | df | *P* |
| --- | --- | --- | --- |
| Isoleucine | 0.014 | 1 | 0.907 |
| N-(2-Furoyl)glycine | 0.364 | 1 | 0.546 |
| Citric acid | 0.023 | 1 | 0.881 |
| Age | 0.047 | 1 | 0.828 |
| Sex | 0.012 | 1 | 0.912 |
| BMI | 1.541 | 1 | 0.215 |
| eGFR | 10.028 | 1 | 0.0015 |
| Global test | 13.328 | 7 | 0.065 |

**Supplementary Table S3.** Sensitivity analysis allowing time-varying effect of eGFR

| Variable | HR (95% CI) | *P* |
| --- | --- | --- |
| Isoleucine | 3.721 (2.272–6.094) | <0.001 |
| N-(2-Furoyl)glycine | 1.655 (1.151–2.380) | 0.0065 |
| Citric acid | 1.527 (1.093–2.133) | 0.013 |
| Age | 1.001 (0.974–1.029) | 0.947 |
| Sex | 1.422 (0.655–3.087) | 0.373 |
| BMI | 1.013 (0.914–1.122) | 0.807 |
| eGFR × log(time+1) | 0.999 (0.993–1.004) | 0.645 |

**Supplementary Table S4.** Bootstrap internal validation of the final multivariable Cox model

| Metric | Value |
| --- | --- |
| Apparent C-index | 0.823 |
| Optimism-corrected C-index | 0.797 |
| Optimism | 0.026 |

Bootstrap validation was performed using 1000 resamples.

**Supplementary Table S5.** Sensitivity analyses of multivariable Cox models assessing the independent association between urinary isoleucine and DKD progression after additional adjustment for storage duration and urine specific gravity

| Model | Variable | HR (95% CI) | *P* |
| --- | --- | --- | --- |
| Model 1 | Isoleucine | 1.949 (1.360–2.794) | <0.001 |
| Model 2 | Isoleucine | 1.933 (1.349–2.768) | <0.001 |
|  | Storage duration (per year) | 0.963 (0.852–1.088) | 0.544 |
| Model 3 | Isoleucine | 1.994 (1.382–2.878) | <0.001 |
|  | Specific gravity (per 0.01 unit) | 0.776 (0.387–1.556) | 0.474 |

Model 1: Base model adjusted for age, sex, BMI, and eGFR.

Model 2: Model 1 additionally adjusted for storage duration.

Model 3: Model 1 additionally adjusted for urine specific gravity.

**Supplementary Table S6.** Characteristics of selected instrumental variables associated with urinary isoleucine levels

| SNP | CHR | EA | OA | MAF | β | SE | *P* | N | F |
| --- | --- | --- | --- | --- | --- | --- | --- | --- | --- |
| rs76372770 | 14 | C | T | 0.102 | 1.621 | 0.317 | 4.16E-06 | 64 | 26.215 |
| rs11617805 | 13 | G | A | 0.367 | -0.987 | 0.197 | 5.98E-06 | 64 | 25.199 |
| rs224619 | 11 | G | A | 0.445 | -0.807 | 0.165 | 9.44E-06 | 64 | 23.920 |
| rs1475514 | 1 | G | A | 0.164 | 1.192 | 0.250 | 1.45E-05 | 64 | 22.752 |
| rs688473 | 10 | C | T | 0.383 | 0.833 | 0.177 | 1.71E-05 | 64 | 22.269 |
| rs2861997 | 3 | G | A | 0.175 | 1.199 | 0.255 | 1.83E-05 | 63 | 22.161 |
| rs2259306 | 7 | C | T | 0.203 | 1.059 | 0.226 | 1.91E-05 | 64 | 21.977 |
| rs10882692 | 10 | C | T | 0.148 | -1.233 | 0.265 | 2.14E-05 | 64 | 21.682 |
| rs12432187 | 14 | G | T | 0.484 | -0.843 | 0.185 | 2.96E-05 | 64 | 20.812 |
| rs602690 | 11 | G | A | 0.359 | 0.971 | 0.214 | 3.28E-05 | 64 | 20.547 |
| rs77889521 | 10 | C | T | 0.055 | -1.611 | 0.356 | 3.28E-05 | 64 | 20.524 |
| rs7960461 | 12 | G | A | 0.109 | -1.405 | 0.313 | 3.77E-05 | 64 | 20.162 |
| rs7841293 | 8 | G | A | 0.056 | -2.034 | 0.454 | 4.14E-05 | 62 | 20.054 |

CHR: chromosome; EA: effect allele; OA: other allele; MAF: minor allele frequency; β: effect size in exposure; SE: standard error; N: sample size; F: F statistic.

**Supplementary Table S7.** Causal effect estimates of urinary isoleucine on kidney disease progression using instrumental variable methods

| MR model | Effect measure | Estimate | 95% CI | *P* | J-test *P* |
| --- | --- | --- | --- | --- | --- |
| TSPS | COR | 2.06 | 1.12–3.81 | 0.021 | - |
| MSMM | CRR | 2.26 | 2.15–2.39 | <0.001 | 0.9999 |

MR: Mendelian randomization; TSPS: two-stage predictor substitution; MSMM: multiplicative structural mean model; COR: causal odds ratio; CRR: causal risk ratio; J-test: Hansen’s test for overidentifying restrictions.
